# Supplementary material for: The interactome of intact mitochondria by cross-linking mass spectrometry provides evidence for coexisting respiratory supercomplexes
Source: Mol Cell Proteomics. 2017 Dec 8;17(2):216–32. doi: 10.1074/mcp.RA117.000470 (PMC5795388; doi:10.1074/mcp.RA117.000470)
Supplement: Supplemental Data [file supp_RA117.000470_133922_0_supp_23978_fzffwf.zip › spectra_annotation/mito_DR_spectra_annotation/213-1-2-1-6-1.pdf]

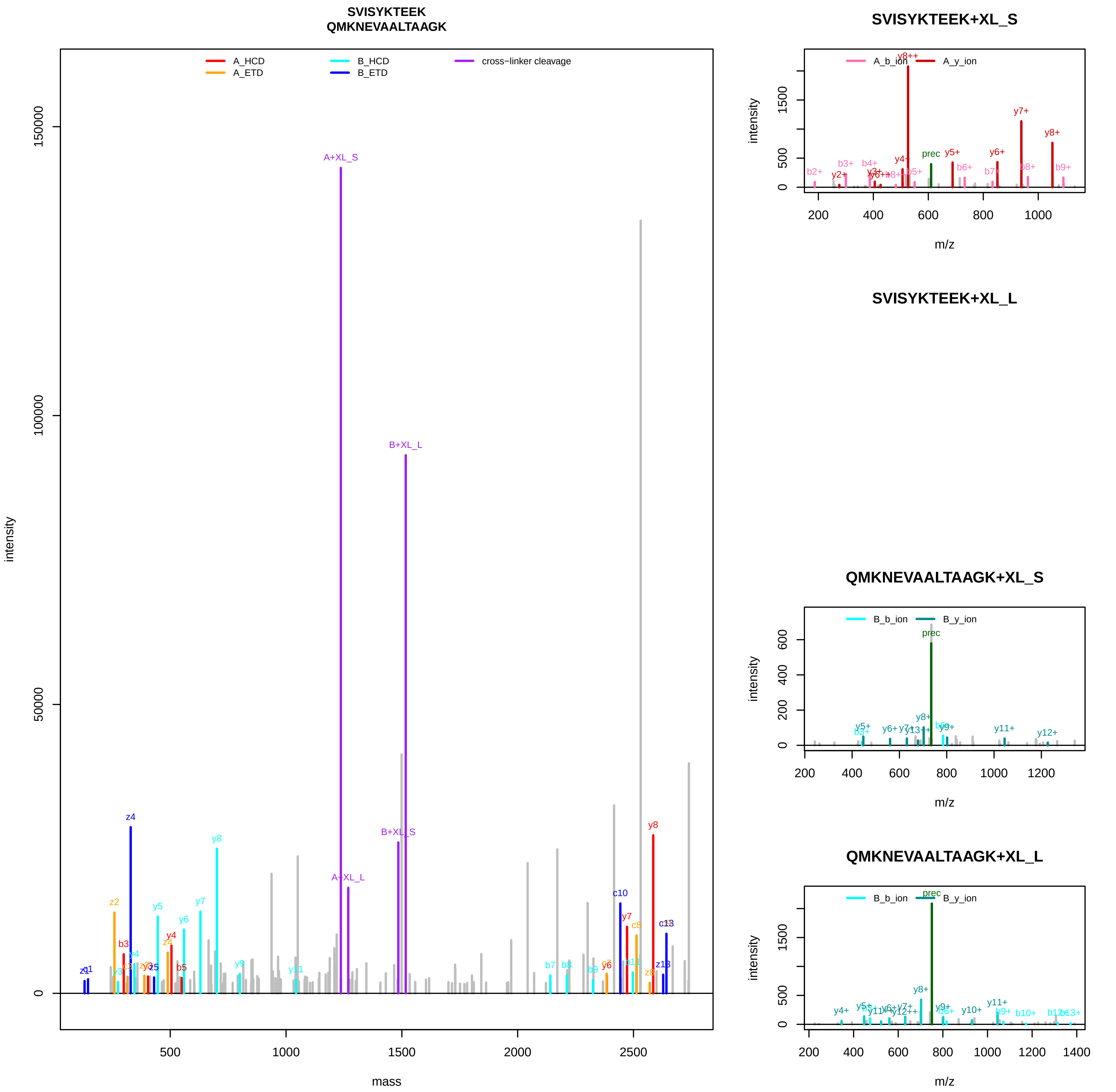
SVISYKTEEK
QMKNEVAALTAAGK
A_HCD
A_ETD
B_HCD
B_ETD
cross−linker cleavage
A+XL_S
B+XL_L
B+XL_S
A+XL_L
intensity
mass
0
50000
100000
150000
500
1000
1500
2000
2500
z4
y8
y7
y5
y6
z2
b3
y4
z4
b5
c10
y7
c8
y8
c13
z13
b7
b8
b9
y6
SVISYKTEEK+XL_S
A_b_ion
A_y_ion
y8++
y7+
y8+
prec
y5+
y6+
intensity
m/z
SVISYKTEEK+XL_L
QMKNEVAALTAAGK+XL_S
B_b_ion
B_y_ion
prec
y11+
y12+
QMKNEVAALTAAGK+XL_L
prec
y8+
y11+
y10+
b10+
